# ETHNIC DIFFERENCES IN HEALTHY LIFE EXPECTANCY IN CHILE

**DOI:** 10.1093/geroni/igad104.1248

**Published:** 2023-12-21

**Authors:** Moises Sandoval, Marcela Alvear Portaccio, Cecilia Albala

**Affiliations:** University of Chile, Santiago, Region Metropolitana, Chile; Departamento Administrativo Nacional de Estadística - DANE, Bogotá, Distrito Capital de Bogota, Colombia; INTA, Universidad de Chile, Santiago de Chile, Region Metropolitana, Chile

## Abstract

Socioeconomic and sex differences in life expectancy (total and healthy) in Chile have been well established. However, the existence of ethnic differences in population longevity is unknown. Thus, we estimate total, healthy, and unhealthy life expectancy among Mapuche (largest indigenous group) and non-Mapuche older adults (≥60 years) in Chile. We estimated abbreviated mortality tables (using indirect demographic methods) and with the EDES health prevalence we applied Sullivan’s method. Disability was defined as the unhealthy state, evaluated through the methodology suggested by Albala et al. (2004). Results show that at age 60 the Mapuche have a life expectancy 7 years lower (26.4 vs. 18.9 years respectively). Although differences diminish with age, Mapuches always have lower total life expectancy. In addition, we detected that the proportion of disability-free life years is higher for non-Mapuche (1.5, 2.0 and 3.7 times higher at 60, 75 and 90 years respectively). In contrast, Mapuches expect to live a higher fraction of their life with disability compared to non-Mapuche (e.g., 84.1% vs. 62.9% at age 80). This is the first study addressing inequities in healthy life expectancy between the Mapuche ethnic group and non Mapuche population, reflected in lower life expectancy, lower healthy life expectancy and higher disabled life expectancy in Mapuche when compared with no Mapuche population.

